# The Philadelphia Medical Journal and the People of the State of New York

**Published:** 1900-09

**Authors:** 


### THE PHILADELPHIA MEDICAL JOURNAL AND THE PEOPLE OF THE STATE OF NEW YORK.

THE organisation of the medical profession of this state expresses the will of the people, as well as the advice and consent of the profession, as the same has been proclaimed at various times since 1760 down to the year 1882. Since the later date a small minority of the profession has failed to concur in the decision of the majority upon a question of the interpretation of a part of the medical law of the state, has seceded from the regularly organised medical societies, general and local, is striving to form rival organisations throughout the state, and the Philadelphia *Medical Journal* seems to be its organ.

Upon two occasions that journal has attacked the mode of medical organisation of this state. After reading the first we characterised the same as "ignorant, illogical and misleading." We have considered the second contribution and would remark that the onslaught now appears vicious and immoral.

Referring to the organisation of the Medical Society of the State of New York, the first article gave the following:

Membership in the State Medical Society is obtained only through the election of a member of a county society or delegate for the annual meeting of the State Society, each county society being entitled to as many delegates as there are assembly districts in the county in which it is located. . . . . It is evident that a glaring fault in this method of representation lies in the fact that a clique in any county society may almost certainly and indefinitely prevent the election of a nominee and it is equally that all the members of any one of the county societies cannot obtain membership in the parent body, no matter how strong their desire may be. . . . . A body thus organised cannot fairly represent the bulk of the profession of the state, nor can it do so until such restrictions to general membership are removed.

We hold that in scientific discussion inaccuracy is of the nature and essence of immorality; that good taste requires of a writer that he be careful of his facts and, further, that when an editor presumes to meddle in the affairs of others that truthfulness is demanded by common decency.

How far the seceder's organ is inaccurate and all the rest of it, the facts in the case will show.

The society in question is composed of delegates, permanent and honorary members. Delegates are sent by county medical societies, medical colleges, incorporated and voluntary medical societies and the Academy of Medicine of New York. The last report of the society shows that in addition to the county medical societies, twenty medical societies and medical institutions are represented by their duly selected delegates.

Perhaps we may suppose that our "has-been-thoroughly-conversant" critic omitted these important institutions for the sake of brevity.

However, our unpardonable sin yet remains. The several bodies which make up the constituency of the state society may only send delegates; not all the members of the faculties of the several medical colleges may be sent at one time, and not all the members of the voluntary medical societies may be so sent; the iron rule of representative delegate attendance is demanded of all alike. This may be all wrong in principle and in practice, but it is quite American.

We do not assume to have peculiar knowledge as to the advantages and purposes of "*les imbéciles.*" We hear they often write and sometimes get printed, but we do not remember to have been particularly edified by their contributions; and, in spite of what they or others may say or write, our present expectation is that so long as we elect to live in this land, we shall hold with the fathers for government by constitution and majority, and in representation by constitutionally selected minority.

When our "cerebration" compels us to declare, that of our fellows none is great or good enough to represent us, we will try to emigrate, and if the police think differently we will go as quietly as we may to the repair-shop where they tinker and sometimes mend think-tanks.

